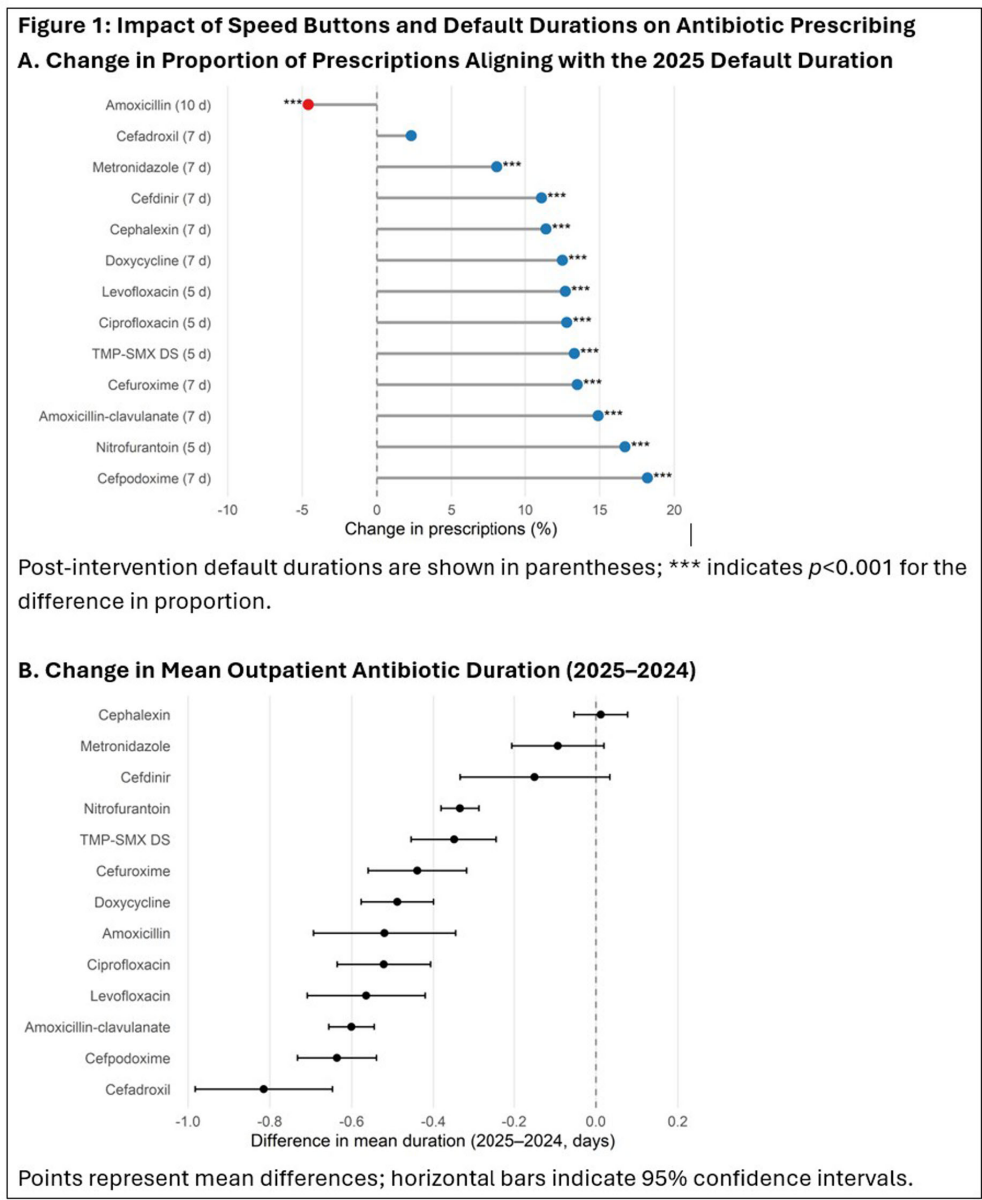# 107 Mortality Rates among Patients Infected with Carbapenem-resistant Acinetobacter baumannii Complex (CRAB), Tennessee, 2014– 2024.

**DOI:** 10.1017/ash.2026.10524

**Published:** 2026-06-23

**Authors:** Noah Boton, Vesna Zunic, Adam Szerencsy, Adrian Tan, Jessica Costella, Dana Mazo

**Affiliations:** 1 NYU Langone Health; 2 NYUPN CIN; 3 NYU Langone health system; 4 NYU Langone

## Abstract

**Background:** A substantial proportion of outpatient antibiotic prescriptions exceed guideline-recommended durations. Although most antibiotic use is in the outpatient setting, the majority of antimicrobial stewardship efforts focus on inpatient care. Prior studies suggest that modifying electronic order defaults is a low-burden strategy to nudge prescribers toward shorter, guideline-concordant courses. We evaluated the impact of updating default durations and adding selectable “speed buttons” to electronic orders for commonly prescribed outpatient antibiotics. **Methods:** We conducted a retrospective pre–post analysis comparing outpatient antibiotic prescriptions during a pre-intervention period (July 1–October 31, 2024) and post-intervention period (July 1–October 31, 2025). Only prescriptions with durations of 1–14 days were included. In June 2025, an electronic order redesign was implemented for the 13 most frequently prescribed outpatient antibiotics, incorporating updated default durations and short-course speed buttons based on guideline-supported treatment ranges for common outpatient infections (Table 1). The primary outcome was the change in proportion of prescriptions aligning with the new default duration. The secondary outcome was the change in mean prescribed duration for each antibiotic. **Results:** There were 80,669 pre-intervention and 86,895 post-intervention prescriptions. All antibiotics except amoxicillin demonstrated an increase in prescriptions aligning with the new default duration (Figure 1A), and all increases were statistically significant except for cefadroxil. The largest increases were observed for cefpodoxime (18.2%) and nitrofurantoin (16.7%). For cefadroxil, the largest absolute shift was a reduction in 10-day courses, decreasing by 10.9% (p<0.001). Amoxicillin defaults remained 10 days in both periods, with new 5-, 7-, and 10-day speed buttons added. Five-day amoxicillin courses increased by 5.5% (p<0.001), representing the largest shift in amoxicillin prescribing. Statistically significant differences in mean prescribed duration were observed for most antibiotics, with small absolute reductions noted (Figure 1B). The largest absolute reductions in mean duration were observed for cefadroxil (−0.82 days) and cefpodoxime (−0.64 days). No significant change was observed for cephalexin, cefdinir, or metronidazole. Cephalexin and metronidazole lacked pre-intervention default durations. **Conclusions:** The addition of short-course speed buttons and updated default durations impacted the length of outpatient antibiotic prescriptions. The intervention increased alignment with guideline-consistent durations and resulted in consistent reductions in mean prescribed duration across most agents. These findings suggest that simple, low-burden changes to electronic antibiotic order design can effectively influence antibiotic prescribing patterns.

**Figure:**
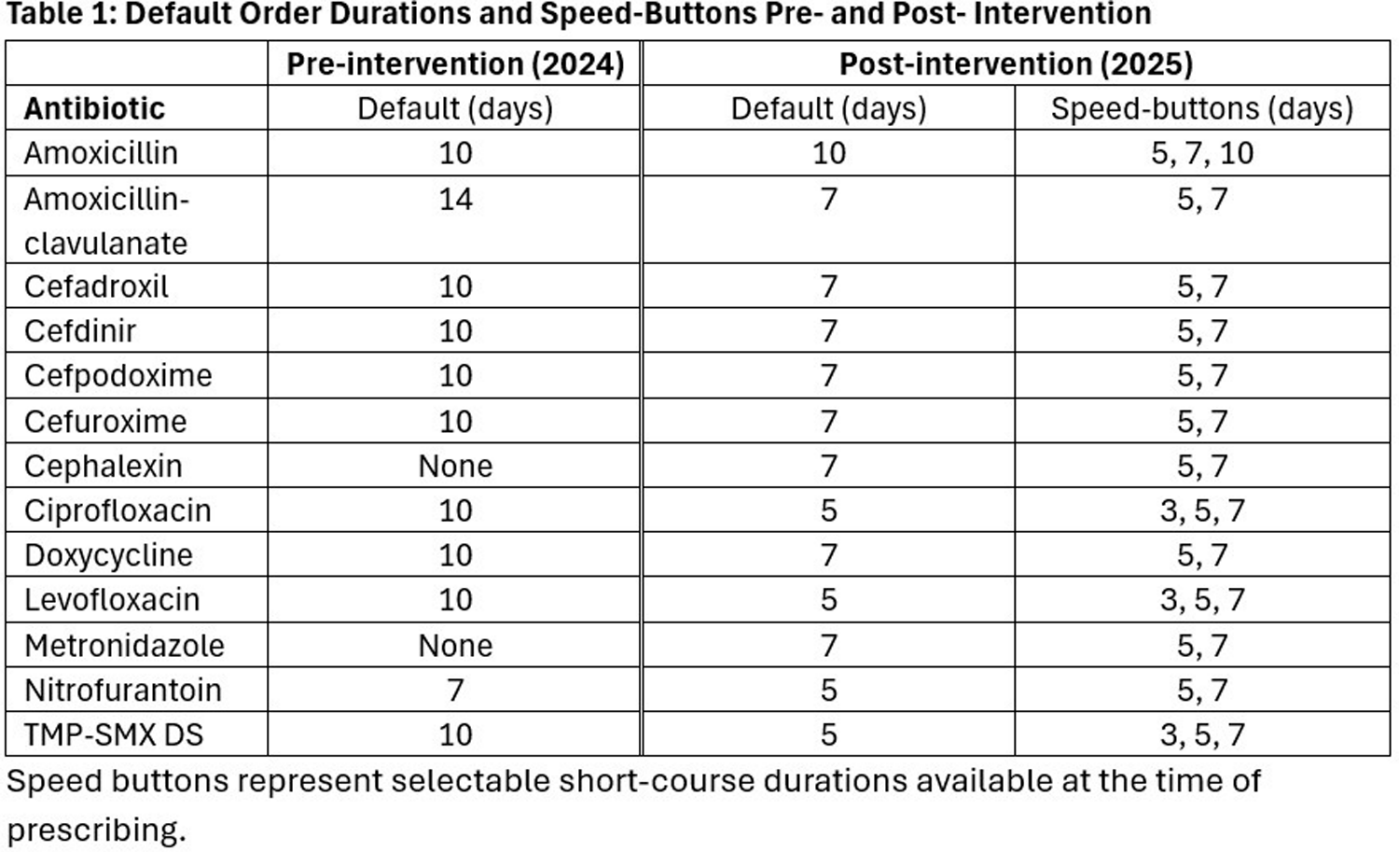

**Figure:**